# Non-invasive volumetric analysis of asymptomatic hands using a 3-D scanner

**DOI:** 10.1371/journal.pone.0182675

**Published:** 2017-08-10

**Authors:** Hiroki Shinkai, Michiro Yamamoto, Masahiro Tatebe, Katsuyuki Iwatsuki, Shigeru Kurimoto, Hitoshi Hirata

**Affiliations:** Department of Hand Surgery, Nagoya University Graduate School of Medicine, Nagoya, Japan; Rush University Medical Center, UNITED STATES

## Abstract

Hand swelling is one of the symptoms often seen in practice, but none of the available morphometric methods can quickly and efficiently quantify hand volume in an objective manner, and the current gold-standard volume measurement requires immersion in water, which can be difficult to use. Therefore, we aimed to analyze the accuracy of using 3-dimensional (3-D) scanning to measure hand volume. First, we compared the hand volume calculated using the 3-D scanner to that calculated from the conventional method among 109 volunteers to determine the reliability of 3-D measurements. We defined the beginning of the hand as the distal wrist crease, and 3-D forms of the hands were captured by the 3-D scanning system. Second, 238 volunteers (87 men, 151 women) with no disease or history of hand surgery underwent 3-D scanning. Data collected included age, height, weight, and shoe size. The wrist circumference (WC) and the distance between distal wrist crease and tip of middle finger (DDT) were measured. Statistical analyses were performed using linear regression to investigate the relationship between the hand volume and these parameters. In the first study, a significantly strong positive correlation was observed [R = 0.98] between the hand volume calculated via 3-D scanning and that calculated via the conventional method. In the second study, no significant differences between the volumes, WC or DDT of right and left hands were found. The correlations of hand volume with weight, WC, and DDT were strong. We created a formula to predict the hand volume using these parameters; these variables explained approximately 80% of the predicted volume. We confirmed that the new 3-D scanning method, which is performed without touching the hand and can record the form of the hand, yields an accurate volumetric analysis of an asymptomatic hand.

## Introduction

Hand swelling is a common symptom of injury or disease, including complex regional pain syndrome, and is important as an indicator of both disease and the effectiveness of treatment [1,2]. In addition, if hand swelling is mild, such as in lymphedema, it is difficult to recognize [3]. Despite the availability of different methods to measure hand volume, none of these morphometric methods can quickly and efficiently quantify hand volume in an objective manner [4,5]. In addition, a standard for healthy hand volume is yet to be defined, and replacing this basic subjective value with an objective one is of great importance.

While several methods of measuring potential hand edema are available, more convenient and accurate methods are needed. The classical volumetric method for measuring hand volume is based on Archimedes’ principle of fluid displacement [4–6]. Other means of estimating hand volume are based on data such as the circumferential measurement method [7].

Using water to measure hand volume has some problems and is not easily practicable. Soaking an injured or painful limb in liquid may cause medical problems such as pain and infection [8]. Using water to measure hand volume is the current gold standard, but is not without issues; the accuracy of measurement may be reduced by a variety of factors [9] including changes in surface tension due to differences in temperature and atmospheric pressure, density of water, and limitations on the accuracy of graduations. Existing tools such as computed tomography (CT) or magnetic resonance imaging (MRI) can measure the volume of body parts; however, CT scanning is associated with radiation exposure [10], and MRI is expensive [11]. In comparison with these tools, 3-D scanning is a rapid, easy, and non-invasive morphometric method for assessing hand volume. Moreover, the cost of 3-D scanning methods will likely be lower than that of MRI because the price of 3-D scanning devices is similar to or lower than that for standard ultrasonography. Measuring the volume of body parts using 3-D scanning has been previously reported [12–15].

The aim of the present study was to examine the reliability of volume assessments of asymptomatic hands using a 3-D scanner. In addition, we sought to define a healthy hand volume. Healthy hand volume is expected to vary with body size, but the morphometric relationship is unclear. While there are few prior studies on potential relationships, a high correlation has been reported between body volume and normal extremity volume [16], suggesting that despite minor fluctuations [17], body measurements may be of value in determining baseline hand volume if an uninjured hand is not available for comparison. Therefore, the relationship of hand volume with age, height, weight, shoe size, and other parameters was investigated.

## Methods

### Participants

The study was conducted from May 2015 to October 2016 following institutional review board approval. Participants were recruited by convenience sampling from the medical staff related with our institution (our institutional ethics board cleared it). After participants were informed consent in written, participants were measured immediately.

First, we performed a study to investigate the utility of 3-D scanning for volume measurement. Using one hand from each of 109 subjects (age range 19–64 years; 63 men and 46 women; 54 right hands and 56 left hands; no history of hand injury or surgery, no symptoms, and no disease under treatment), we compared conventional and 3-D scanning methods for both accuracy and reliability. Scanning, processing and analysis of image data was performed by the same examiner. The conventional measurement method entailed immersion of the participant’s hand into a vessel filled with water up to the distal wrist crease, and measuring the change in water volume [4, 5]. This procedure was repeated ten times for one hand. The standard deviation was 3.7 ml.

To control for inter- and intra-tester correlations, two examiners (A, B) scanned the same hand three times and the volume of the hand was measured according to the protocol described below. These examiners were hand surgeons and medical staff, informed in general use of the device, but not provided advanced technical training.

Next, a total of 238 participants (87 men, age range 22–62 years; 151 women, age range 17–66 years; no history of hand injury or surgery, no symptoms, and no disease under treatment) agreed to participate in a second study. Data collected for each participant included age (years), height (cm), weight (kg), shoe size (cm), and hand dominance. Shoe size was self-reported. Scanning, processing and analysis of image data was performed by the same examiner. We investigated whether these items can be correlated with or predictive of hand volume using multiple regression analysis.

### Methodology of 3-D imaging and measurement

**Fig 1:**
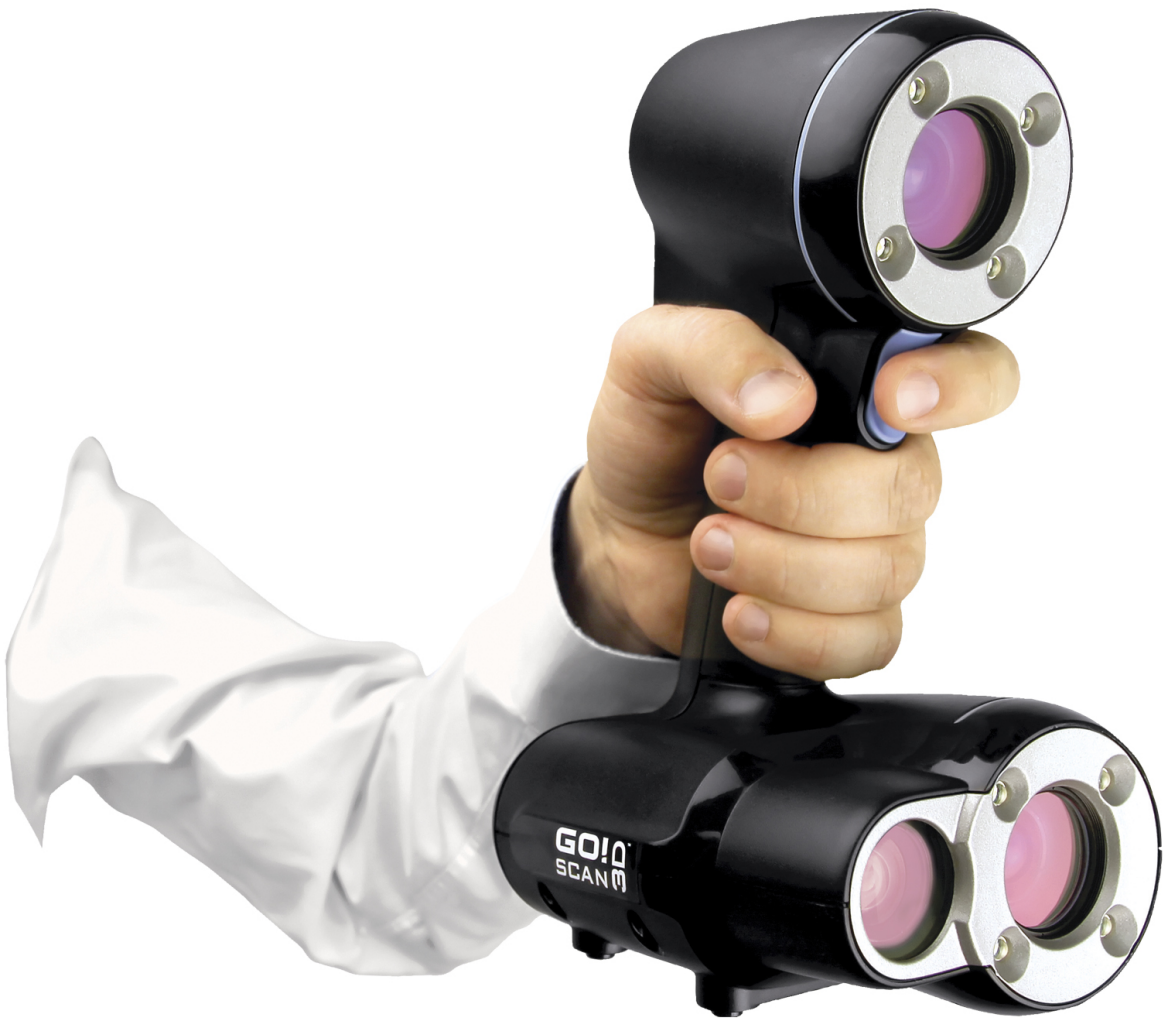
The 3-D scanning system. This device (GO!SCAN, CREAFORM [AMETEK group] Québec, Canada) is of convenient size, and can capture 3-D images by lighting the object directly and detecting reflections. This image was provided by the company.

Hand volume was measured using a 3-D scanning system with a manufacturer-reported precision of 0.5 mm (Fig 1) [18]. 3-D scanning involves allowing lighting the object and then detecting its reflection. Participants removed hand accessories, rested their elbows on a table and placed their hands in the neutral position of the wrist and elbow joint. They then opened their hands and maintained their fingers at full extension during the scanning. The examiner manipulated the 3-D scanner to illuminate the entire circumference of the hand capturing its image data. The same examiner performed 3-D imaging of all participants in addition to processing the resulting 3-D images.

### Three-dimensional image rendering process

**Fig 2:**
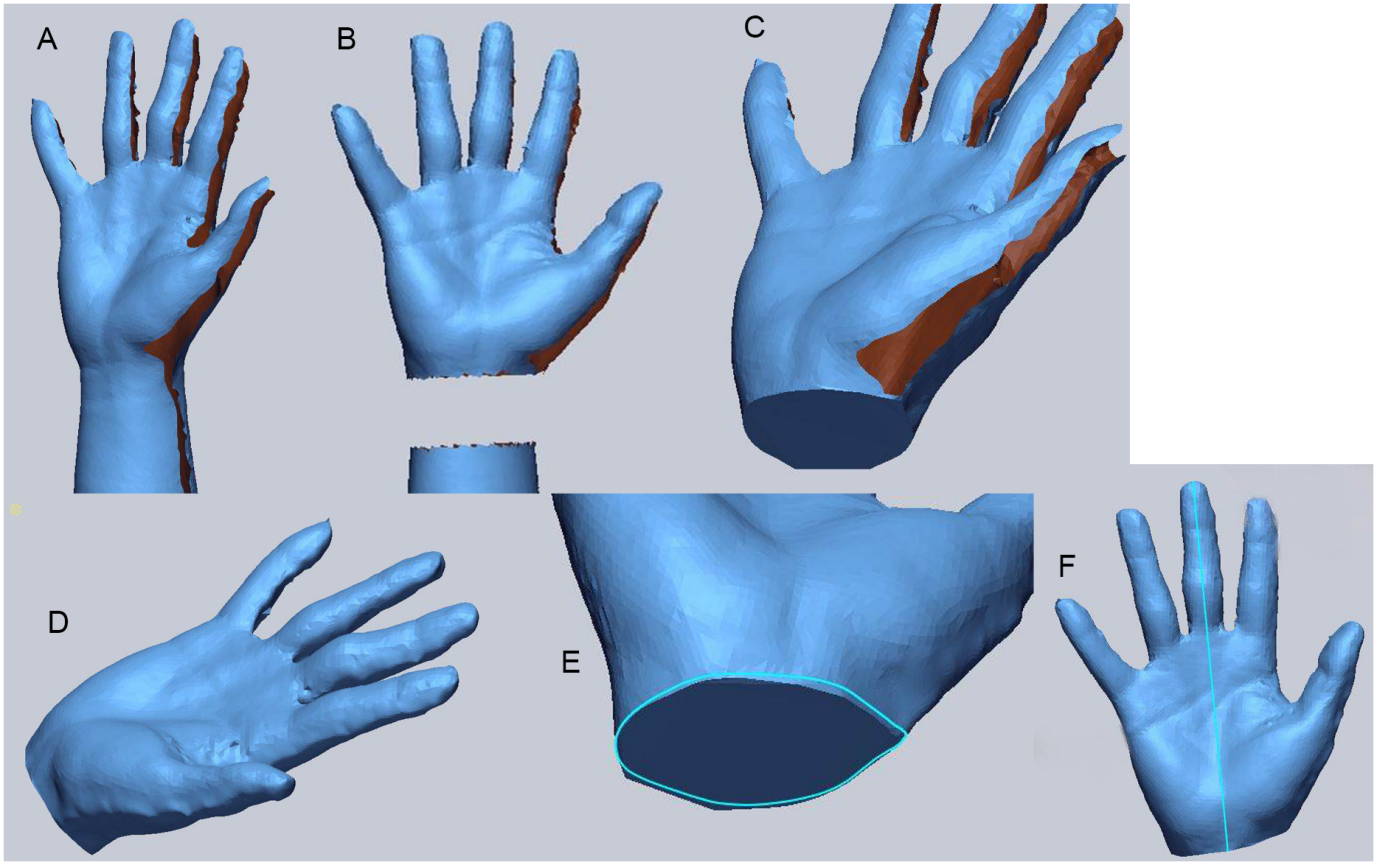
Three-dimensional imaging. Geomagic Verify software was used (3D Systems Inc., South Carolina, USA). The raw scan was saved as a single surface (A). The 3-D image was cropped at the distal wrist crease (B), and the cut plane was filled (C). The gaps observed in the 3-D image were filled using the application’s ‘bridge’ and ‘filling’ tools (D). The blue line shows the wrist circumference (E). The blue line shows the distance between the distal wrist crease and tip of middle finger (F).

The rendering process created a smooth surface profile using Geomagic Verify software (Fig 2) [19]. The raw scan was saved as a single surface, not in contact with any objects, with the hand fixed. The 3-D image was cropped at the distal wrist crease perpendicular to the long axis of the forearm. The obtained 3-D images exhibited partial loss of data because the light did not reach certain areas, such as the web, or due to light over-reflection from other areas, such as the nail. These areas were not detected by the device. In order to measure the volume, these missed areas in the 3-D image were filled using the application’s ‘bridge’ and ‘filling’ tools. Artifacts were removed from the raw scan data. The software calculated the volume of the 3-D hand model and measured the wrist circumference (WC) and the distance between distal wrist crease and tip of middle finger (DDT) using the calculated volume.

### Statistical analysis

First, a paired *t-*test was performed to assess the significance of differences between the water displacement method and 3-D scanning method. Linear regression analysis was then performed to determine the correlation coefficient of the two methods. A paired *t-*test was used to check for significant differences between the parameters of the right and left hands. There were two women, whose scanned data was lost, therefore these cases were excluded in the 3-D analyses, but not in the baseline parameters. To investigate possible correlations between hand volume and other parameters, the Pearson correlation coefficient was computed and a linear regression analysis was performed. Bland-Altman analysis was performed to determine systematic bias and limits of agreement (LOA). To analyze the intra- and inter-class correlation coefficients (ICC), a reliability analysis was performed. Statistical significance was set to a level of *P* < 0.05 for all two-tailed tests.

## Results

### Accuracy of 3-D scanning

**Fig 3:**
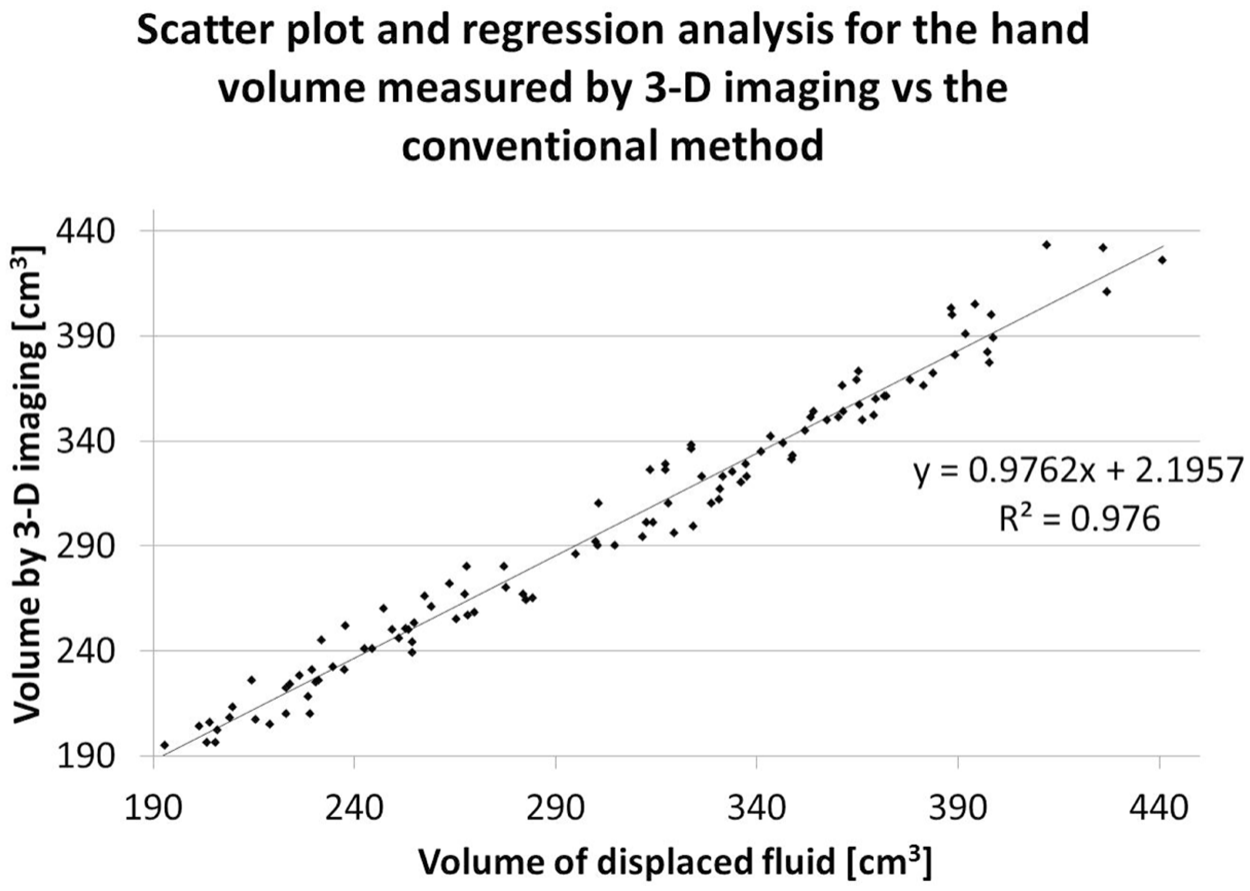
Scatter plot with regression analysis. The average hand volume of 109 hands measured using 3-D imaging is plotted against the volume measured using the method based on Archimedes’ principle. A regression line was generated from these plotted points, with a coefficient of determination (R) of 0.98.

Comparing the values obtained from traditional and 3-D scanning methods, a strong positive correlation was observed [R = 0.98] (Table 1, Fig 3). The slope of the equation is close to 1 and the intersection is close to zero. This result showed that hand volume measured by the new 3-D scanning method was highly correlated with the volume measured using the conventional method. According to the Bland-Altman analysis, proportional error was not found (r = 0.07), and fixed bias was recognized (95% confidence interval did not contain 0). LOA was between -14.6 and 24.6 ml (% LOA = -4.3% to 7.2%).

**Table 1:** Comparing the values obtained from traditional and 3-D scanning.

			Men	Women	*p*-value
***N***		109	63	46	
**Age, years**		38.7 ±10.8	36.9 ±9.9	41.3 ±11.7	
**Hand, n**	**Right**		31	23	
	**Left**		32	23	
**Hand volume**	**Traditional method, cm**^**3**^		298.3 ±64.3		<0.01
	**3-D scanning, cm**^**3**^		303.3 ±65.1		

The volumes for both right and left hands were combined. cm^3^, cubic centimeter.

The inter- and intra-tester variability was minimal, as shown in Table 2. The results of ICC analysis indicated that a single measurement by the 3-D imaging method was sufficient to obtain an accurate hand volume value. The reliability and reproducibility of 3-D volumetric measurement were not affected by the examiner.

**Table 2:** The inter- and intra-rater variability.

Examiner	ICC (1,1)	95% CI	ICC (2,1)	95% CI
**A**	0.99	0.95–0.99	0.99	0.98–0.99
**B**	0.99	0.98–0.99		

ICC, intra-class correlation coefficient; CI, confidence interval

### Three-dimensional scanning parameters

Baseline characteristics of the male and female participants are shown in Table 3. The demographic parameters include two women who were excluded from the 3-D analysis because their left hand data was lost. In both men and women, the volumes of the right and left hands were not significantly different (p = 0.21 and 0.30, respectively). In addition, in both, WC and DDT were not significantly different between the right and left hands (WC: p = 0.40 and 0.83; DDT: p = 0.42 and 0.38, respectively) (Table 4).

**Table 3:** Baseline characteristics of participants.

		Men (n = 87)	Women (n = 151)
**Age, mean (SD), years**		38.1 (9.5)	38.0 (11.1)
**Age group, n**	20–29	21	44
	30–39	34	45
	40–49	23	39
	50–59	21	21
	60–69	2	2
**Mean BMI (SD), kg/m**^**2**^		22.8 (3.0)	20.9 (3.6)
**Mean shoe size (SD), cm**		26.3 (1.0)	23.5 (2.1)
**Dominant hand, n**	Right	83	141
	Left	3	2
	Cross-dominant	1	8

BMI, body mass index; SD, standard deviation; kg/m^2^, kilogram per square meter.

**Table 4:** Measured parameters.

		Hand volume, cm^3^	WC, mm	DDT, mm
**Men**	**Right (SD), n = 87**	354.2 (42.4)	169.7 (8.5)	197.4 (9.6)
	**Left (SD), n = 87**	346.1 (42.6)	168.6 (9.0)	198.5 (9.1)
	***P*-value**	0.21	0.40	0.42
**Women**	**Right (SD), n = 151**	249.2 (36.9)	153.9 (15.0)	180.0 (16.5)
	**Left (SD), n = 149**	244.7 (37.5)	153.6 (15.1)	180.8 (16.6)
	***P*-value**	0.30	0.83	0.38

The results for women include two participants whose left hand scanned data was lost, hence they are included only in the volume data, and not in the other parameter analyses. Consequently, for the scanned parameters: n = 149 instead of n = 151 in this analysis only for the left hand side. Hand volume obtained using 3-D scanner. cm^3^, cubic centimeter; WC, wrist circumference; DDT, distance between distal wrist crease and tip of the middle finger, SD, standard deviation.

### Regression analysis for hand volume

**Fig 4:**
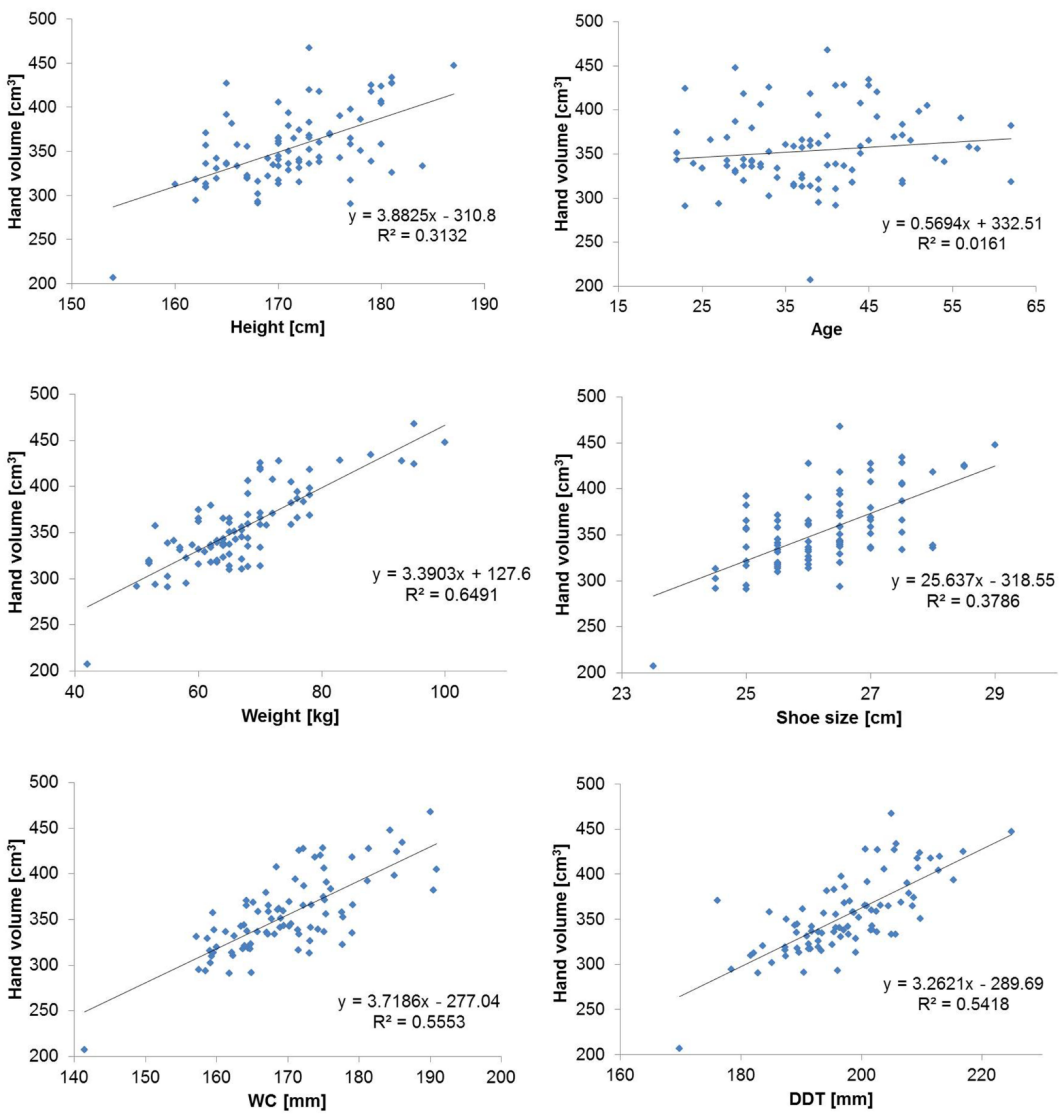
Scatter plots with linear regression of right hand volume against different parameters in men (*N* = 87). The hand volume as measured using 3-D imaging was plotted against each parameter (age, height, weight, shoe size, WC, and DDT). A regression line was generated from the plotted points, and a coefficient of determination was calculated for each plot. Compared to women, the correlation of body weight and DDT with hand volume was larger. WC, wrist circumference; DDT, distance between the distal wrist crease and the tip of the middle finger.

**Fig 5:**
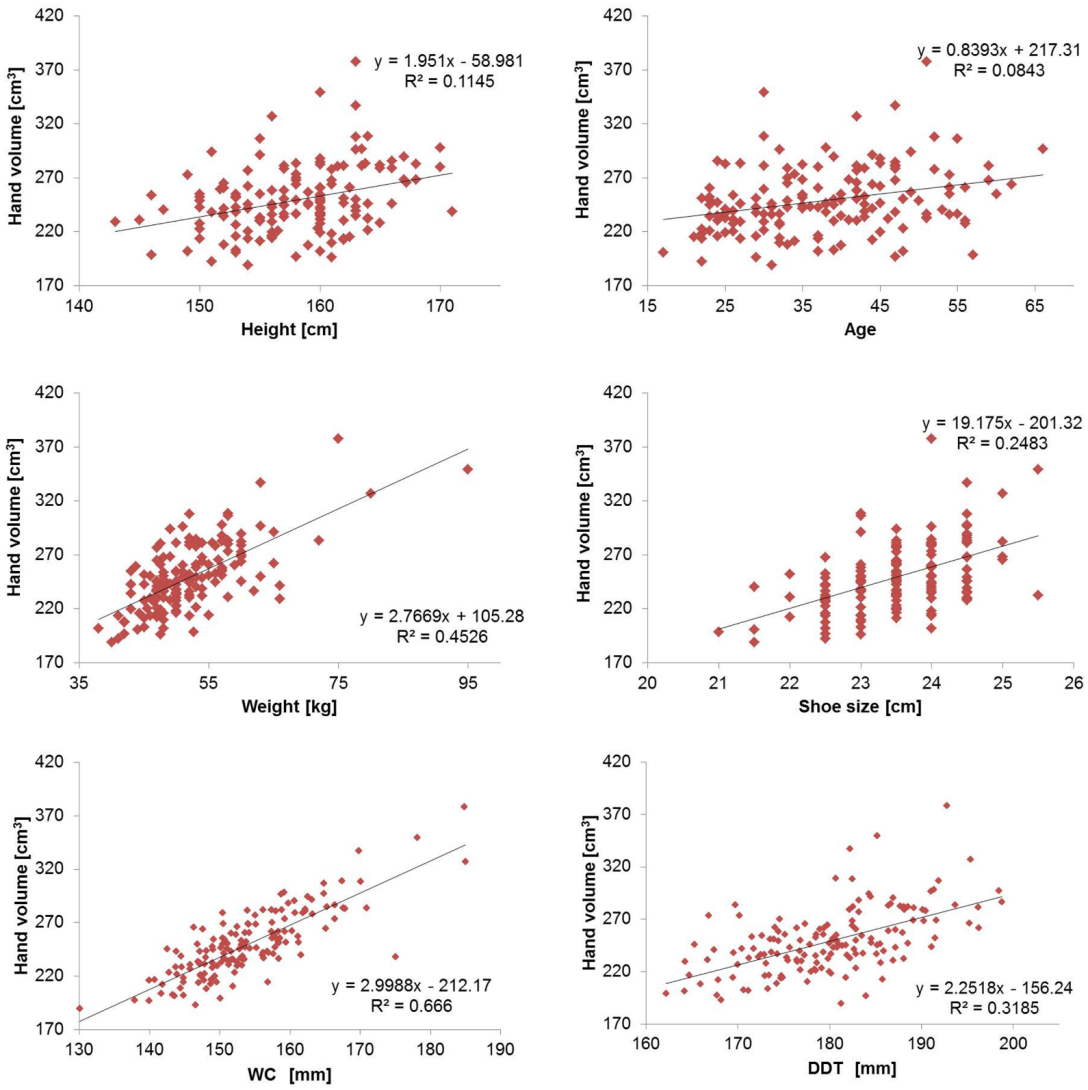
Scatter plots with linear regression of right hand volume in women (N = 151) against different parameters. The hand volume measured using 3-D imaging was plotted against each parameter (age, height, weight, shoe size, WC, and DDT). A regression line was generated from the plotted points, and a coefficient of determination was calculated for each plot as shown. Compared to men, the correlation of body weight and DDT with hand volume was smaller. WC, wrist circumference; DDT, distance between the distal wrist crease and the tip of the middle finger.

Linear regression analysis was performed to assess the relationship between right hand volume and other parameters (Figs 4 and 5). Only the results of the right hand are shown because there was no significant laterality.

In men and women, the correlations between hand volume and age, height, and shoe size were weak, whereas strong positive correlations were found between hand volume and weight, WC, and DDT. These results were the same for the left and right hands. Multiple regression analysis was performed using weight, WC, and DDT as explanatory variables due to their strong positive correlations with hand volume (Table 5, data shown for the right hand only). Based on the results of multiple regression analysis, the correlation coefficients and determination coefficients of hand volume estimated from WC, DDT, and body weight were calculated (Table 6, data shown for the right hand only). A total of 79–82% of the predicted hand volume in men and women could be explained by a combination of weight, WC, and DDT.

**Table 5:** Multiple regression analysis results.

Right hand		β	95% CI	*P*-value	VIF	r^2^ vs hand volume
**Men** **(*N* = 87)**	**Weight, kg**	0.44	1.29–2.43	<0.0001	2.85	0.65
	**WC, mm**	0.23	0.48–1.84	0.001	2.25	0.56
	**DDT, mm**	0.39	1.24–2.23	<0.0001	2.18	0.54
**Women** **(*N* = 151)**	**Weight, kg**	0.21	0.47–1.33	<0.0001	1.83	0.45
	**WC, mm**	0.56	1.64–2.44	<0.0001	2.99	0.67
	**DDT, mm**	0.32	0.95–1.60	<0.0001	1.47	0.32

Hand volume obtained using 3-D scanner. VIF, variance inflation factor; r^2^, coefficient of determination; WC, wrist circumference; DDT, distance between distal wrist crease and tip of the middle finger.

**Table 6:** Multiple regression analysis using DDT, weight, and WC.

	R	R^2^	Modified R^2^	Formula for calculating the hand volume
**Men**	0.90	0.82	0.81	-309.39+1.86×(Weight)+1.73×(DDT)+1.16×(WC)
**Women**	0.89	0.79	0.78	-346.06+0.88×(Weight)+1.31×(DDT)+2.04×(WC)

Hand volume obtained using 3-D scanner. WC, wrist circumference; DDT, distance between distal wrist crease and tip of the middle finger.

## Discussion

Volumetric analysis of the hand using a 3-D scanner is non-invasive, quick, and easy, and has accuracy comparable to that of the classical method. Our study aimed to find way to measure asymptomatic hand volume using a 3-D scanner. The method of volume measurement using this device was reliable, and has the potential to be a useful clinical tool [12].

Using the water displacement method to measure hand volume can be technically difficult. Immersion of a hand in a water tank can also be risky and uncomfortable for a patient with an injured hand. Accuracy is dependent on attention to detail and the size of the water tank; small changes in angulation of the hand can also lead to error [9]. However, if the measurement environment is well controlled, the error is about 1% [20]. Therefore, the water displacement method is currently regarded as the gold standard for measuring the volume of body parts [21]. We investigated the consistency of measurements taken using 3-D scanning with those taken using water displacement in this study. Although a very high correlation between the conventional method and 3-D scanning method was observed, the %LOA was -4% to 7%, compared to a %LOA of water displacement of −2.6% to 1.4% [22]. While the 3-D scanning method was less accurate than the water displacement method, the error caused by the 3-D scanning method is considered to be clinically acceptable given the advantages over water displacement. The 3-D scanning method can store data as a shape rather than a numerical value, revealing the location as well as the extent of subsequent swelling. In addition, although we did not record measurement time per hand in this study, the process can be completed in less than 10 minutes. Touching the hands is not necessary for measurement, which can be important in the case of injury.

Previous studies have reported on physical volume measurement using imaging technologies such as CT and MRI [10,11]. The use of non-invasive morphological evaluation using a 3-D scanner has been increasing recently, and the technique has been used mostly to analyze surface anatomy [23,24]. Although several studies have evaluated hand volume measurement using a 3-D scanner [22, 25], an attempt has not yet been made to define a standard for the normal value or to predict healthy hand volume from existing measurements. Volume estimation is subject to errors related to the necessary approximations in the volume-calculation formula, which can be especially important when measuring the volume of swollen hands [26].

In the second study, we measured different parameters related to hand volume using the 3-D scanner, and used these parameters to formulate an equation to predict the asymptomatic hand volume. We found that approximately 80% of the hand volume could be explained by weight, WC, and DDT. While these parameters do not predict the hand volume accurately enough to be useful in the clinic for all patients, the predicted hand volume derived from this formula could help inform estimates of the severity of edema if an unaffected hand is not available for comparison.

WC, DDT, and the formula to predict the hand volume would not be needed when the hand volume could be derived from a volumetric analysis using the 3-D scanner. However, the formula has two possible uses. First, we may predict the hand volume without the 3-D device using the formula. The WC and DDT calculated by the software are almost identical to actual data. Thus, in the cases of systemic diseases or bilateral injuries, in which the contralateral part does not yield what can be considered a normal value, the DDT and WC (if not affected by swelling) could be used for estimation of the normal volume. Second, if it is possible to measure the 3-D hand volume, we may recognize hand swelling quantitatively by comparing the actual or 3-D hand volume to the hand volume calculated from the actual WC and DDT if there is a clear dissociation in both numerical values. In such cases, the cause of hand swelling may be checked using ultrasound devises or other imaging modalities.

While the determination coefficient of 0.8 was statistically significant, this study targeted only asymptomatic hands, and the accuracy has not been sufficiently verified for clinical use. The present result suggested that further research is needed to increase the accuracy of the method.

The results of this study showed the feasibility of using the non-invasive 3-D scanner to measure hand volume. Although the basic data collected regarding hand volume were limited and the variability of the measurements was wide (warranting more data collection), it may be possible to determine the range for asymptomatic hand volume using 3-D scanning. This 3-D volumetric analysis has not yet been applied to swollen hands. The accuracy of 3-D scanning to determine changes in volume of injured or swollen hands needs to be evaluated; however, now that the 3-D volumetric analysis method has been established, we will be able to quantitatively compare the volume of patients’ swollen hands to that of their normal hands.

External validity testing was not performed for the formula used to predict the hand volume from weight, WC and DDT. The use of this formula is still limited clinically; however, it can be used to predict the volume of asymptomatic hands and to track volume changes in the same hands. Evaluating hand swelling may be possible after collecting more hand measurements and different body parameters. This formula could become a tool to aid in diagnostic and treatment decisions for swollen hands.

There are some limitations to this study. These included the use of the distal wrist crease to define the hand. The definition of the distal wrist crease as the beginning of the hand is controversial. In patients with an unclear crease due to obesity or age, or other pathological changes, relying on this defining point is fundamentally problematic. It may be more accurate to identify a boundary line based on bone. However, superficial landmarks are much more easily utilized in clinical practice, and using one was consistent with our study goals. We found the distal wrist crease to be a clear and stable landmark in all hands.

All the participants were Japanese, and we did not consider physical differences. The distribution of normal hand volumes varied widely, and there was a possibility that some participants had unknown inflammatory conditions that may have affected hand volume. The predicted hand volume using our formula might deviate from actual volume if we forcibly fit the outliers to the formula. In addition, this 3-D technique is suited for defining the surface anatomy; thus, error may be attributed to hand position, diurnal variation, or to the method of measurement, resulting in difficulties in complete quantification. While all images were processed by the same person, the reliability of image processing was not evaluated.

## Conclusion

Volumetric analysis of the hand using 3-D scanning provides accuracy equal to that of the classical method and has the potential to be easily utilized. Strong correlations between certain parameters and hand volume, which may aid in estimating healthy hand volume, were also revealed in our research.

## Supporting information

S1 Table Data sets of 3-D scanning parameters. These data sets are original data of the baseline characteristics (Table 3) and the measurement parameters (Table 4).
(XLSX)
